# Epigallocatechin Gallate Potentiates the Anticancer Effect of *AFP*-siRNA-Loaded Polymeric Nanoparticles on Hepatocellular Carcinoma Cells

**DOI:** 10.3390/nano14010047

**Published:** 2023-12-23

**Authors:** Kamonlatth Rodponthukwaji, Ponpawee Pingrajai, Saranrat Jantana, Seri Taya, Kongpop Duangchan, Kytai T. Nguyen, Chatchawan Srisawat, Primana Punnakitikashem

**Affiliations:** 1 Department of Biochemistry, Faculty of Medicine Siriraj Hospital, Mahidol University, Bangkok 10700, Thailand; kamonlatth.rod@mahidol.ac.th (K.R.); saranrat.jant@gmail.com (S.J.); seritaya12@gmail.com (S.T.); kongpop.dua@student.mahidol.ac.th (K.D.); chatchawan.sri@mahidol.ac.th (C.S.); 2 Research Network NANOTEC-Mahidol University in Theranostic Nanomedicine, Faculty of Medicine Siriraj Hospital, Mahidol University, Bangkok 10700, Thailand; ponpawee.pin@gmail.com; 3 Siriraj Center of Research Excellence in Theranostic Nanomedicine, Faculty of Medicine Siriraj Hospital, Mahidol University, Bangkok 10700, Thailand; 4 Department of Bioengineering, University of Texas at Arlington, Arlington, TX 76019, USA; knguyen@uta.edu

**Keywords:** poly(lactic-co-glycolic) acid, epigallocatechin gallate, siRNA, liver cancer

## Abstract

To develop a potential cancer treatment, we formulated a novel drug delivery platform made of poly(lactic-co-glycolic) acid (PLGA) and used a combination of an emerging siRNA technology and an extracted natural substance called catechins. The synthesized materials were characterized to determine their properties, including morphology, hydrodynamic size, charge, particle stability, and drug release profile. The therapeutic effect of *AFP*-siRNA and epigallocatechin gallate (EGCG) was revealed to have remarkable cytotoxicity towards HepG2 when in soluble formulation. Notably, the killing effect was enhanced by the co-treatment of *AFP*-siRNA-loaded PLGA and EGCG. Cell viability significantly dropped to 59.73 ± 6.95% after treatment with 12.50 μg/mL of EGCG and *AFP*-siRNA-PLGA. Meanwhile, 80% of viable cells were observed after treatment with monotherapy. The reduction in the survival of cells is a clear indication of the complementary action of both active EGCG and *AFP*-siRNA-loaded PLGA. The corresponding cell death was involved in apoptosis, as evidenced by the increased caspase-3/7 activity. The combined treatment exhibited a 2.5-fold increase in caspase-3/7 activity. Moreover, the nanoparticles were internalized by HepG2 in a time-dependent manner, indicating the appropriate use of PLGA as a carrier. Accordingly, a combined system is an effective therapeutic strategy.

## 1. Introduction

Cancer is among the leading causes of death worldwide, including lung, colon, breast, and liver cancers. Over 830,000 deaths caused by liver cancer were reported in 2020, which represented the third most common cause of mortality [1]. According to this number, scientists from the International Agency for Research on Cancer (IARC) and partner institutions have just predicted that new cases and deaths caused by liver cancer will increase by 55% in 2040 [2]. Although there are several potential treatments, such as surgery, radiation, and chemotherapy, adverse effects can still be found and cause harm to patients. For example, chemotherapy destroys adjacent healthy tissues as much as tumors [3,4,5]. Particularly, hepatocellular carcinoma (HCC), the most prevalent form of primary liver cancer, has shown resistance to chemotherapy drugs [6].

Due to side effects caused by chemotherapy, biological substances have emerged as an interesting and alternative option for cancer treatments. Epigallocatechin-3-gallate (EGCG), an active polyphenol ingredient in green tea, has demonstrated anticancer effects by inhibiting tumor growth through cell cycle arrest and apoptosis induction [7,8,9]. An effective inhibition induced by EGCG was also elicited in HCC by showing an IC50 of 60–80 µM [10,11]. Besides EGCG, other polyphenol compounds found in green tea such as caffeic acid (CA); gallic acid (GA); catechin (C); epicatechin (EC); gallocatechin (GC); catechin gallate (CG); gallocatechin gallate (GCG); epicatechin gallate (ECG); and epigallocatechin (EGC) also demonstrated anticancer effects including antiproliferation, cell cycle arrest, and cell apoptosis. Still, EGCG was the most potent active compound compared to the others [9].

In addition, small interfering RNA (siRNA), an interesting therapeutic option, has been reported as a powerful tool for cancer treatment over recent years. In the advanced stage of HCC, it can be diagnosed through the measurement of a biomarker called serum alpha-fetoprotein (AFP). In HCC patients, high AFP levels are detected [12]. Thus, siRNA targeting the *AFP* gene, designated as *AFP*-siRNA, has become one of the most promising strategies for inhibiting *AFP* expression. Reportedly, silencing of *AFP* can be achieved in the hepatoma cell line HepG2 by reducing *BIRC5* (survivin) mRNA expression [13]. Also, the expression of *AFP* was completely inhibited in the overexpressed *AFP* hepatocellular carcinoma cell line (EGHC-9901) by the *AFP*-siRNA plasmid transfection system through apoptosis [14]. Moreover, the decrease in *AFP* expression could be promoted by knocking down fibroblast growth factor receptor 4 (FGFR4) through MAPK signaling in the S2 cell line [15]. Nevertheless, the delivery of siRNA alone into the cytosol of the cells could be an issue due to its negative charge and ease of degradation. To achieve the highest therapeutic effects, exploiting nanoparticles as siRNA carriers could efficiently render the loaded cargo to the target without degradation [16].

Nanotechnology has been used as a drug delivery system for decades. A variety of materials have been extensively studied, such as mesoporous silica nanoparticles [17], carbon nanotubes [18], liposomes [19], and polymer-based materials [20,21]. PLGA nanoparticles have been widely chosen as drug carriers owing to their biocompatibility, biodegradability, tunability in size, and prolonged circulation time. Most importantly, PLGA biomaterial was approved by the Food and Drug Administration (FDA) for clinical use [22]. Many studies revealed the successful therapeutic efficacy of siRNA-encapsulated PLGA-based delivery platforms. For example, PLGA-based nanoparticles (NPs) encapsulated with *BIRC5*-siRNA displayed promising anticancer properties as they could decrease tumor size and survivin expression [23]. In our previous study [24], we successfully generated *AFP* siRNA-loaded PLGA nanoparticles, which could suppress *AFP* expression and specifically induce apoptosis in HepG2 cells.

The efficacy of EGCG in inhibiting tumor proliferation through cell cycle arrest and apoptosis and the capacity of *AFP*-siRNA in suppressing cell growth by interfering in cell growth processes could be hampered due to the rapid degradation of siRNA and toxicity caused by high-dose usage of EGCG. Therefore, we developed a biocompatible polymeric nanocarrier system for delivering siRNA to the target site. Along with the effective siRNA-nanocarrier system, combination treatment with EGCG would possibly enhance the anticancer effects. Here, the physical properties and the ability of the polymeric nanoparticles as carriers were first investigated. Then, a comprehensive study regarding the function of *AFP*-siRNA in inhibiting cell proliferation via a cell viability assay was performed. In addition, the synergistic anticancer effects of *AFP*-siRNA and active EGCG were also investigated through a cell viability assay and caspase-3/7 activity in the system with or without the aid of PLGA nanoparticles.

## 2. Materials and Methods

### 2.1. Chemicals and Biological Reagents

All chemicals and reagents were used as received without further purification. Chloroform (≥99.8%), poly(lactic acid-co-glycolic) acids (carboxylic acid end group PLGAs) with MW of 100,000 g/mol, epigallocatechin gallate (EGCG), and dimethylsulfoxide (DMSO) were purchased from Sigma-Aldrich (St. Louis, MO, USA). Lipofectamine^®^ 2000 Transfection Reagent and Block-iT^TM^ Alexa Fluoro^®^ Red Fluorescent Oligo were bought from Invitrogen (Carlsbad, CA, USA). Alpha-fetoprotein siRNA (*AFP*-siRNA) and scrambled (Scr)-siRNA were obtained using GenePharma (Suzhou, China). CellTiter-Blue^®^ Cell Viability Assay, Apo-ONE^®^ Homogeneous Caspase-3/7 Assay, and QuantiFluor^®^ RNA system were acquired from Promega (Madison, WI, USA). Modified Eagle Medium (DMEM), fetal bovine serum (FBS), and penicillin–streptomycin (Pen-Strep) were received from Gibco (Grand Island, NY, USA).

### 2.2. Synthesis of siRNA-Loaded PLGA Nanoparticles

siRNA-loaded nanoparticles were synthesized using a standard emulsion method described previously with some modifications [25,26]. Firstly, the solution of siRNA (siScr or *AFP*-siRNA) at the desired amount was added dropwise into the stirring solution of PLGA containing 3% *w*/*v* of PLGA in chloroform. Next, the mixed solution was sonicated by using an ultrasonic probe sonicator (Sonics and Materials Inc., Newtown, CT, USA) with 30% amplitude. Then, the first emulsion containing siRNA and PLGA was constantly dropped into a 5% polyvinyl alcohol (PVA) solution. After that, the nanoparticle suspension was sonicated and subsequently stirred at room temperature overnight to remove chloroform. Finally, the particles, siRNA-PLGA NPs, were obtained using a Sorvall RC6+ centrifuge (Thermo Fisher Scientific, Asheville, NC, USA) at 15,000 rpm at 4 °C for 30 min and resuspended in nuclease-free water. Lyophilized particles could also be obtained. Scrambled (Scr)-siRNA served as a control. An unloaded PLGA had the same protocol carried out on it without adding a solution of siRNA. For particle tracking in the cellular internalization study, the same protocol for siRNA-loaded PLGA was applied using Block-iT^TM^ Alexa Fluoro^®^ Red Fluorescent Oligo (BLOCK-iT) as a representative of *AFP*-siRNA. The percentage of encapsulation efficiency was determined with a direct method by dissolving *AFP*-siRNA-loaded PLGA with DMSO and determining the loaded *AFP*-siRNA content using a QuantiFluor^®^ RNA system according to the manufacturer’s protocol. The % encapsulation efficiency can be calculated by following the below equation.

$$\mathrm{Encapsulation}\;\mathrm{efficiency}\;\left(\%\right)=\left[\frac{\mathrm{loaded}\;\mathrm{amount}\;\mathrm{AFP}\text{-}\mathrm{siRNA}}{\mathrm{added}\;\mathrm{amount}\;\mathrm{AFP}\text{-}\mathrm{siRNA}}\right]\times 100$$

### 2.3. Characterization of the Synthesized Nanoparticles

The hydrodynamic size and zeta potential of the fabricated nanoparticles were measured using a Zetasizer (Malvern Instruments, Worcestershire, UK). The morphology was determined by field emission scanning electron microscopy (FESEM) (SU8030, Hitachi, Ltd., Tokyo, Japan). The stability study was performed by measuring the hydrodynamic size change over a 5-day period when incubating these NPs in PBS solution and DMEM containing 10% FBS solution at pH 7.4. The percentage encapsulation efficiency of the payload (%EE) was attained by the direct method, as described in the previous section. The amount of loaded siRNA was correlated to the fluorescence intensity measured at Ex480/Em530 nm. The fluorescence intensity was obtained by using Multi-Detection Microplate Readers (Bio-Tek Instrument Inc., Winooski, VT, USA). The drug release profile of *AFP*-siRNA-loaded PLGA was determined using the centrifugation method. The released media were replaced with fresh media of the same volume and the amount of loaded siRNA was determined using the QuantiFluor^®^ RNA system.

### 2.4. Cell Viability and Caspase-3/7 Activity

The HepG2 cell line was purchased from the Cell Lines Service (Eppelheim, Germany). The cells were cultured in Dulbecco’s Modified Eagle Medium (DMEM) supplemented with 10% fetal bovine serum (FBS) and 1% penicillin-streptomycin at 37 °C, 5% CO_2_, in an incubator. Cell viability was determined by CellTiter-Blue^®^ Cell Viability Assays (Promega, Madison, WI, USA). Briefly, HepG2 cells (1 × 10^4^ cells/well) were seeded in a 96-well plate and incubated at 37 °C, 5% CO_2_, in an incubator. Then, the cells were treated with the compounds of interest (siRNA, siRNA-loaded nanoparticles, and EGCG) and incubated at 37 °C, 5% CO_2_, in an incubator for 24 h. After that, the fluorescence signals were measured at Ex545/Em590 nm using Multi-Detection Microplate Readers (Bio-Tek Instrument Inc., Winooski, VT, USA). For the caspase-3/7 activity assay, a consecutive protocol was performed after the cell viability assay. The mixture of caspase-3/7 substrate and buffer was prepared according to the manufacturer’s protocol for the Apo-ONE^®^ Homogeneous Caspase-3/7 Assay (Promega, Madison, WI, USA), and was then added into each well at a volume ratio of 1:1 of the CellTiter-Blue and caspase-3/7 mixture. The mixture was then incubated in the dark for 30 min at 37 °C. The fluorescence intensity was measured at Ex485/Em530 nm using Multi-Detection Microplate Readers (Bio-Tek Instrument Inc., Winooski, VT, USA).

### 2.5. Cellular Internalization

HepG2 (1 × 10^5^ cells/well) was seeded in a 24-well plate and incubated at 37 °C, 5% CO_2_, in an incubator for 24 h. Then, the studied material of BLOCK-iT-loaded PLGA nanoparticles was added into the cells and incubated at 37 °C, 5% CO_2_, for 3 h and 6 h. Then, the transfected cells were collected and determined using a BD FACS Celesta™ Cell Analyzer (BD Bioscience, San Jose, CA, USA).

### 2.6. Statistical Analysis

All results were presented as mean ± SD (*n* = 3). The statistical analysis of multiple-group comparisons was tested by one-way ANOVA followed by Tukey’s post hoc test using IBM SPSS statistics, Version 28.0 (IBM Corp., Armonk, NY, USA).

## 3. Results and Discussion

### 3.1. Physical Characterization of the Synthesized Nanoparticles

**Scheme 1:**
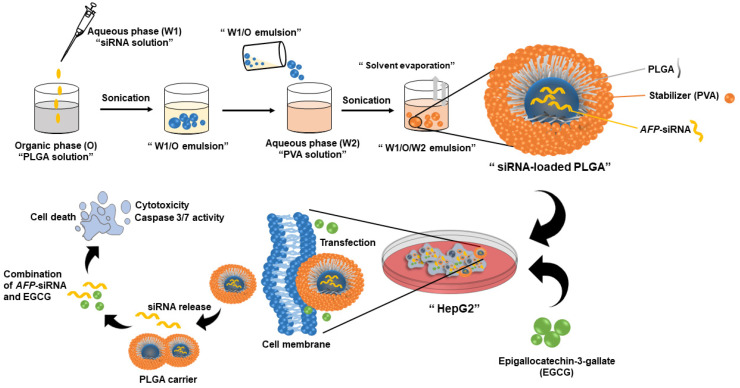
Schematic representation of the overall concept of the therapeutic delivery system.

A combined anticancer effect of a natural active ingredient, EGCG, along with an *AFP*-siRNA-loaded biocompatible drug carrier (PLGA nanoparticles), has been first investigated in this work. A therapeutic delivery system for the *AFP*-siRNA-loaded PLGA nanoparticles combined with EGCG was successfully fabricated. The schematic representation of the synthetic method and its biological study is displayed in Scheme 1.

**Figure 1:**
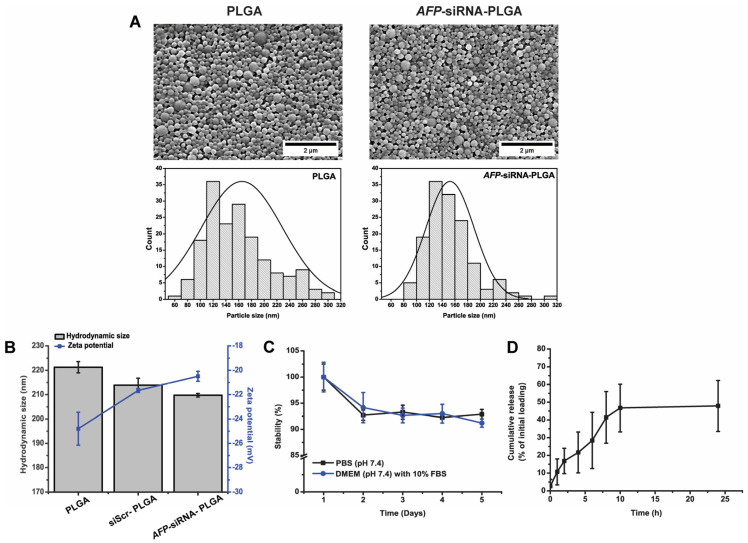
Nanoparticle characterization: (**A**) Morphology of unloaded PLGA and *AFP*-siRNA PLGA nanoparticles characterized by FESEM and their size distributions of both nanoparticles determined from FESEM. (**B**) Hydrodynamic size and zeta potential of the synthesized PLGA, siScr-PLGA, and *AFP*-siRNA-PLGA. (**C**) Stability test over a 5-day period of siScr-PLGA nanoparticles in two different environments, PBS (pH 7.4) and DMEM medium containing 10% FBS at 37 °C using Zetasizer. (**D**) Drug release profile of *AFP*-siRNA-loaded PLGA over 24 h. The presented data are mean ± SD (*n* = 3).

The morphology and physical property of the materials were characterized using field emission scanning electron microscopy (FESEM) and a Zetasizer, respectively. The morphology of unloaded PLGA and *AFP*-siRNA-loaded PLGA nanoparticles is displayed in Figure 1A. The unloaded PLGA revealed a spherical shape with a smooth surface. The particles were quite monodispersed in size and shape. The size distribution obtained from FESEM showed a mean particle size of 164.79 ± 60.34 nm. However, loading *AFP*-siRNA inside the PLGA core did not affect the morphology of the particles. The same observation was noticed when an anticancer drug called DOX was packed inside the PLGA co-delivery system [27]. However, after loading siRNA, the *AFP*-siRNA-PLGA demonstrated a slight reduction in size with a narrower size distribution (152.40 ± 37.64 nm) than the unloaded one.

The hydrodynamic diameters of nanoparticles were analyzed using a dynamic light scattering method. Figure 1B illustrates the hydrodynamic sizes of unloaded PLGA, scrambled siRNA (siScr)-PLGA, and *AFP*-siRNA-PLGA nanoparticles. Unloaded PLGA demonstrated a hydrodynamic size of 221.31 ± 2.31 nm, while a slight decrease in size was observed in scrambled siRNA (siScr)-PLGA (213.90 ± 2.85 nm) and *AFP*-siRNA-PLGA (209.73 ± 0.72 nm), suggesting no effect on nanoparticle formation with and without drug loading. This observation is also consistent with previous works regarding oligonucleotide-loaded PLGA nanoparticles [28,29]. The slight reduction in size of siRNA-loaded PLGA determined by DLS was also consistent with the observation from FESEM images. This effect might be attributed to the greater stabilization of the inner aqueous phase (W1) of siRNA-PLGA than the unloaded one. Feczkó et al. [30] demonstrated that a solution of bovine serum albumin that was dissolved in phosphate-buffered saline (PBS) could affect the final particle size, although it resided in the innermost phase. Thus, the inner droplets possibly influenced the final particle size. This observation could be supported by Tu’s work [31]. W1, with the presence of PBS, which we used to solubilize siRNA in this work, can maintain the pH of the inner phase and ionize the carboxylic acid end group of PLGA, thus facilitating the interaction of PLGA at the oil-water interface and enhancing the stabilization of double-emulsion nanoparticles afterwards. Also, the heterogeneity presented as the polydispersity index (PI) of the synthesized *AFP*-siRNA-PLGA nanoparticles still showed shallow values of 0.04, suggesting high particle dispersibility in the studied environment without particle aggregation [32]. In addition, the unloaded PLGA nanoparticle showed a negative charge of −24.80 ± 1.35 mV. After payload loading, the siScr-PLGA and *AFP*-siRNA-PLGA still revealed a negative charge of −21.66 ± 0.16 mV and −20.49 ± 0.41 mV, respectively, confirming no effects on their physical properties after loading with siRNA.

Moreover, the stability of the synthesized particle was also monitored by measuring the hydrodynamic size under two different environments, PBS (pH 7.4) and DMEM medium containing 10% FBS. Herein, siScr was used as a representative of *AFP*-siRNA. According to Figure 1C, the result illustrated only a slight decrease in the stability of the siScr-PLGA nanoparticles in both environments over a 5-day incubation time at 37 °C. This negligible change in size could be due to the degradation of PLGA through hydrolysis, which typically occurred very quickly at an initial time and followed by a slow degradation afterwards, leading to a noticeable decrease in size at the first 24 h [33]. However, despite the presence of serum, the polymeric nanoparticles were still well dispersed in the mimicking biological system due to the existence of the stabilizer, PVA, preventing aggregation [34]. Also, the incorporated PVA in the structure may help the nanoparticles avoid opsonization in clinical applications [35]. This result implied that the synthesized nanocarrier has good stability. The PLGA-based nanoparticles were even stable in a prolonged period of up to 2 weeks in previous studies [36].

To fulfil its ability as a carrier, the encapsulation efficiency of the payload was also determined. In this work, the *AFP*-siRNA encapsulation efficiency was 42.22 ± 7.35%. Our fabricated polymeric nanoparticles showed a similar efficiency for entrapping the payload to one previously reported [37]. In addition, the capability of releasing the loaded therapeutic, *AFP*-siRNA, was also studied and is revealed in Figure 1D. Regarding the result, it could be observed that the PLGA behaved with a burst release character in the first 10 h by releasing about 46.73 ± 13.44% of *AFP*-siRNA. The gradual release was continued and showed about 47.92 ± 14.37% release at 24 h, similar to previous work [38]. This sustained release character of the synthesized material made them feasible for being a good drug delivery system. Regarding the results from materials characterization, our nanoparticles provided appropriate physical properties in terms of stability, efficient drug encapsulation, and prolonged drug release. Thus, it could be determined that the synthesized PLGA was a suitable candidate for the siRNA delivery system.

### 3.2. Anticancer Effects of AFP-siRNA and EGCG

**Figure 2:**
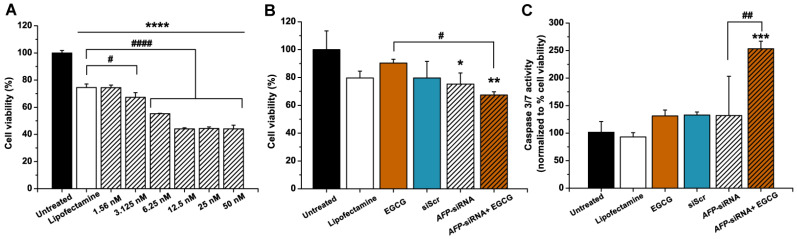
The biological effect of the therapeutic agents towards HepG2 cells at 24 h: (**A**) The cytotoxicity of transfected *AFP*-siRNA at different concentrations by using lipofectamine 2000 as a transfection reagent. (**B**) The combination effect of *AFP*-siRNA (3.125 nM) and EGCG (25 μg/mL) toward cell viability. (**C**) The caspase-3/7 activity of the cells after treatment with siScr, *AFP*-siRNA, EGCG, and combined *AFP*-siRNA and EGCG. The presented data are mean ± SD (*n* = 3). A significant difference between the sample and untreated cell control is statistically identified with *: *p* < 0.05, **: *p* < 0.01, ***: *p* < 0.001, ****: *p* < 0.0001. A significant difference between two samples is statistically identified with #: *p* < 0.05, ##: *p* < 0.01, ####: *p* < 0.0001.

Firstly, the antiproliferative effect of siRNA was assessed by using lipofectamine 2000 as a transfection reagent. The concentration of transfected *AFP*-siRNA varied from 1.56 nM to 50 nM in a two-fold-increasing manner. The cell cytotoxicity of the *AFP*-siRNA assessed using the CellTiter-Blue^®^ Cell Viability Assay is illustrated in Figure 2A. To verify the effect of siRNA alone, lipofectamine 2000 served as a transfection reagent and was used as a control group. As a result, the cell viability of HepG2 after incubation with the transfected siRNA for 24 h was decreased in a dose-dependent manner. Notably, at a concentration of 1.56 nM of *AFP*-siRNA, it did not show any toxicity to the cells compared to the lipofectamine control group. A significant increment in cell death was observed when 3.125 nM of *AFP*-siRNA was used, leaving 67.32 ± 3.38% of viable cells. When a higher dosage of transfected *AFP*-siRNA (at 6.25 nM) was applied, the percentage of surviving cells was decreased to 55.22 ± 0.34%. However, these survival cell numbers reached their maximum when a higher content of siRNA was applied (i.e., 12.5 nM to 50 nM). As HepG2 is a representative of *AFP* mRNA-expressing cell lines, a significant drop in cell survival in this work was likely due to the ability of transfected *AFP*-siRNA in silencing *AFP* mRNA. Since the suppression of *AFP* mRNA was correlated to the inhibition of cell proliferation [39], the cascade event of cell viability was then observed. This result is also consistent with the previous report from our group [40]. Thus, it could be confirmed that the cell death was undoubtedly attributed to the effect of *AFP*-siRNA, exhibiting potential as an anticancer therapeutic agent.

Furthermore, to determine the anticancer effect of combining two active therapeutic agents, *AFP*-siRNA and EGCG, cell viability was primarily studied. Figure 2B demonstrated that a single treatment of transfected *AFP*-siRNA at a concentration of 3.125 nM showed slight toxicity to the HepG2 cells compared to untreated cells. A percentage of viable cells of about 75.23 ± 7.97% could be observed. This result ensured the cytotoxicity effect of *AFP*-siRNA as transfected siScr did not exhibit toxicity to the cells. Also, significant cell death could not be observed when a single treatment of 25 µg/mL of EGCG was applied. In contrast to single-drug treatment, transfection of *AFP*-siRNA followed by treatment with EGCG obviously displayed a significant drop in the percentage of cell viability, exhibiting 67.35 ± 2.34% of cell survival. An insight into the cell death mechanism was also revealed in Figure 2C, and it was found that a combination of transfected *AFP*-siRNA and EGCG could cause cell death through caspase-3/7 activity. The acquired caspase-3/7 activity is inversely proportional to the cell viability shown in Figure 2B. A small dose of 3.125 nM of *AFP*-siRNA combined with 25 µg/mL of EGCG could promote caspase-3/7 activity, which is reflected in cell death via the apoptosis pathway [41]. According to the above results, it was likely that a single treatment at low dose levels of either siRNA or EGCG might not show an adequate capability of inhibiting cancer proliferation. Therefore, the use of siRNA technology in combination with an active natural ingredient could be an alternatively attractive platform to improve anticancer treatment.

### 3.3. Anticancer Effects of AFP-siRNA-Loaded PLGA

**Figure 3:**
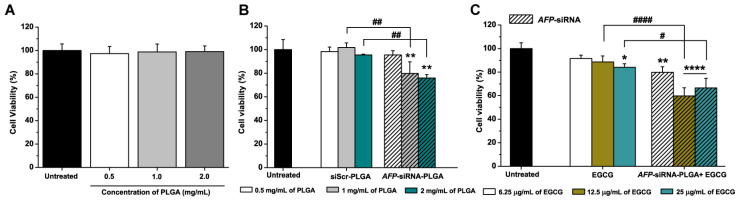
The effect of a synthesized nanoparticle-based delivery system towards HepG2 cells at 24 h: (**A**) Cell viability after treatment with PLGA at different concentrations: 0.5 mg/mL, 1 mg/mL, and 2 mg/mL. (**B**) The effect of loaded siRNA in particulate form on the cell viability at different concentrations of PLGA-loaded nanoparticles: 0.5 mg/mL, 1 mg/mL, and 2 mg/mL. (**C**) The effect of a single treatment of EGCG or a combined treatment of *AFP*-siRNA-PLGA and EGCG on cell viability at various concentrations of ECGC (6.25 µg/mL, 12.5 µg/mL, and 25 µg/mL). The presented data are mean ± SD (*n* = 3). A significant difference between the sample and untreated cell control is statistically identified with *: *p* < 0.05, **: *p* < 0.01, ****: *p* < 0.0001. A significant difference between two samples is statistically identified with #: *p* < 0.05, ##: *p* < 0.01, ####: *p* < 0.0001.

Although we found that *AFP*-siRNA transfected by lipofectamine 2000 could efficiently show an anticancer effect in vitro, its therapeutic effect may not reach efficacy due to the off-target and loss of function of the therapeutics during blood circulation. Establishing an efficient delivery system, by exploiting nanotechnology, would improve the anticancer effect by reducing such adverse effects. Along with suitable physical properties in terms of the stability and drug release behavior of PLGA nanoparticles, the biocompatibility of PLGA was also an important indicator of being a good siRNA carrier. Figure 3A revealed the cytotoxicity of unloaded PLGA to the cells. Although a high concentration of up to 2 mg/mL of nanoparticles was applied, no toxicity was observed. This finding strongly confirmed the biocompatibility of the material.

Consequently, an effective amount of *AFP*-siRNA was loaded in PLGA, and it still showed an antiproliferative effect by reducing the cell viability in a dose-dependent manner, as shown in Figure 3B. A significant drop in cell viability was observed when 1 mg/mL of *AFP*-siRNA-loaded nanoparticles was used, showing a cell viability of 79.76 ± 9.86% in comparison with the siSCR-PLGA counterpart. The reduced cell viability was certainly attributed to the presence of *AFP*-siRNA, as the siSCR-PLGA serving as a control group did not cause a decline at the same concentration of nanoparticles. This was evident in the fact that the therapeutic effect of *AFP*-siRNA remained active in spite of the particulate formulation. The reduction in cell viability observed in this work is consistent with the previous work from our group, confirming the correlation between cell death and *AFP* mRNA suppression by using an *AFP*-siRNA-targeted gene [24]. Although the percentage of cell death observed using the particulate form of siRNA is less than that of the one transfected by lipofectamine 2000, this effect of utilizing nanoparticles as a carrier is compromised by the long-term effect of protecting the payload from degradation. Also, other types of nanomaterials, such as lipid nanoparticles, have been recently focused upon for siRNA delivery. However, the material production is costly and an extremely positive charge of the lipid formulation may cause toxicity to the cells [42]. Due to the high biocompatibility of PLGA, an effective siRNA delivery platform could be successfully established in this work.

### 3.4. Anticancer Effects of AFP-siRNA-Loaded PLGA Combined with EGCG

Next, we explored the effect of the combination of siRNA-loaded PLGA and EGCG on cancer cells. The anticancer effect could be enhanced when an active anticancer compound, EGCG, was used in combination with the constructed *AFP*-siRNA-loaded PLGA. Figure 3C presented an improved antiproliferative impact of cancer cells when EGCG was combined with *AFP*-siRNA-PLGA. The effective doses of EGCG were about 12.5 µg/mL and 25 µg/mL, showing cell viabilities of 59.73 ± 6.95% and 66.58 ± 8.13%, respectively. Meanwhile, cell viability was more than 80% when a single treatment of EGCG at all concentrations was applied. The evidence of diminished cell viability due to the combined treatment in Figure 3C compared to the single therapy confirmed that our constructed therapeutic platform is effective even though the application was a low dose of 12.5 µg/mL of EGCG. This might be due to the sensitization of the cells by *AFP*-siRNA-PLGA, rendering the cells susceptible to angiogenesis inhibitors, as similarly observed in previous work from our group [40].

### 3.5. Cellular Internalization of PLGA

**Figure 4:**
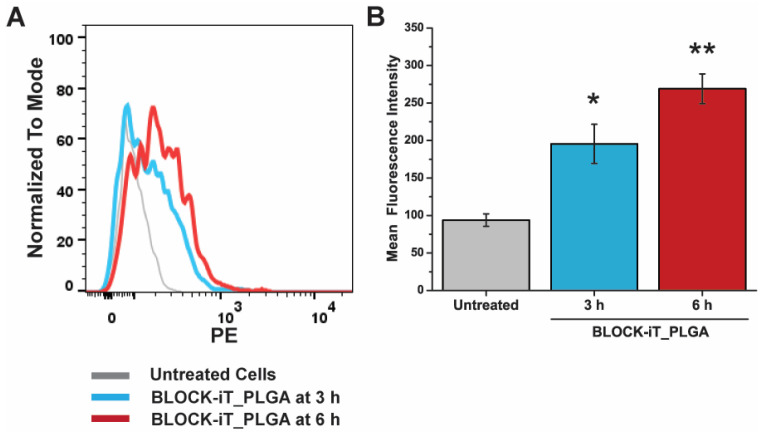
Cellular internalization of BLOCK-iT-loaded PLGA nanoparticles: (**A**) flow cytometry analysis of HepG2 after treating with BLOCK-iT_PLGA at 3 h and 6 h, and (**B**) mean fluorescence intensity analyzed from flow cytometry. The presented data are mean ± SD. A significant difference between the sample and untreated cell control is statistically identified with *: *p* < 0.05, **: *p* < 0.01.

We also investigated the capability of the constructed material, PLGA, in cellular internalization. The PLGA nanoparticle was observed at 3 h and 6 h using flow cytometry. Herein, BLOCK-iT was chosen as the fluorescent siRNA probe for tracking the PLGA nanoparticles in the cellular uptake investigation. According to the histogram graph of the flow cytometry analysis depicted in Figure 4A, it was found that the BLOCK-iT-loaded PLGA nanoparticle could be engulfed into the HepG2 cells with % positive cells of 16.9% and 30.75% at 3 h and 6 h, respectively. Figure 4B shows mean fluorescence intensities of 195.5 and 269.0 at 3 h and 6 h, respectively, which are consistent with the percentage of positive cells. The cellular internalization of nanoparticles in HepG2 occurred in a time-dependent manner, which is consistent with other work [43]. With respect to the size of the nanomaterials, the synthesized material is in the suitable size range to be internalized by the cells. Sun et al. [44] demonstrated the size-dependent effect of PLGA nanoparticles on cellular internalization. The small size of 200 nm was found to be easier to be taken up by the cells compared to the larger ones (500 nm and 2000 nm). Although the travel speeds inside the cells were similar in all size ranges, the larger nanoparticles were retained for a longer time in the cell membrane compared to the small ones. Therefore, our synthesized PLGA can be a suitable material to use as a therapeutic carrier.

## 4. Conclusions

In summary, the synthesized PLGA nanocarriers, along with the therapeutic effects of siRNA and EGCG, have shown remarkable potential as an anticancer drug delivery system. The powerful tool of the anticancer effect of siRNA accompanied with an active ingredient from a natural product can considerably enhance anticancer performance by significantly decreasing the cell viability of cancerous cells compared to single treatment with either siRNA or EGCG alone. Furthermore, this study also emphasized that combining those two therapeutics could cause cell death through the apoptosis pathway. Therefore, this established therapeutic platform is a good candidate for anticancer treatment and is worthy of further investigation to acquire comprehensive data in other aspects of biological studies.

## Data Availability

The data presented in this study are available within the article.
